# Characterization and Survival of Human Infant Testicular Cells After Direct Xenotransplantation

**DOI:** 10.3389/fendo.2022.853482

**Published:** 2022-03-10

**Authors:** Danyang Wang, Simone Hildorf, Elissavet Ntemou, Lihua Dong, Susanne Elisabeth Pors, Linn Salto Mamsen, Jens Fedder, Eva R. Hoffmann, Erik Clasen-Linde, Dina Cortes, Jørgen Thorup, Claus Yding Andersen

**Affiliations:** ^1^ Laboratory of Reproductive Biology, University Hospital of Copenhagen, Rigshospitalet, Copenhagen, Denmark; ^2^ Department of Clinical Medicine, University of Copenhagen, Copenhagen, Denmark; ^3^ Department of Pediatric Surgery, University Hospital of Copenhagen, Rigshospitalet, Copenhagen, Denmark; ^4^ Centre of Andrology & Fertility Clinic, Department D, Odense University Hospital, Odense C, Denmark; ^5^ Research Unit of Human Reproduction, Institute of Clinical Research, University of Southern Denmark, Odense, Denmark; ^6^ Danish National Research Foundation (DNRF) Center for Chromosome Stability, Department of Cellular and Molecular Medicine, Faculty of Health and Medical Sciences, University of Copenhagen, Copenhagen, Denmark; ^7^ Department of Pathology, University Hospital of Copenhagen, Rigshospitalet, Copenhagen, Denmark; ^8^ Department of Pediatrics and Adolescent Medicine, Copenhagen University Hospital Hvidovre, Copenhagen, Denmark

**Keywords:** spermatogonial stem cell, gonocyte, spermatogonia, transplantation, cryptorchid, immature testis, infertility

## Abstract

**Background:**

Cryopreservation of prepubertal testicular tissue preserves spermatogonial stem cells (SSCs) that may be used to restore fertility in men at risk of infertility due to gonadotoxic treatments for either a malignant or non-malignant disease. Spermatogonial stem cell-based transplantation is a promising fertility restoration technique. Previously, we performed xenotransplantation of propagated SSCs from prepubertal testis and found human SSCs colonies within the recipient testes six weeks post-transplantation. In order to avoid the propagation step of SSCs *in vitro* that may cause genetic and epigenetic changes, we performed direct injection of single cell suspension in this study, which potentially may be safer and easier to be applied in future clinical applications.

**Methods:**

Testis biopsies were obtained from 11 infant boys (median age 1.3 years, range 0.5-3.5) with cryptorchidism. Following enzymatic digestion, dissociated single-cell suspensions were prelabeled with green fluorescent dye and directly transplanted into seminiferous tubules of busulfan-treated mice. Six to nine weeks post-transplantation, the presence of gonocytes and SSCs was determined by whole-mount immunofluorescence for a number of germ cell markers (MAGEA, GAGE, UCHL1, SALL4, UTF1, and LIN28), somatic cell markers (SOX9, CYP17A1).

**Results:**

Following xenotransplantation human infant germ cells, consisting of gonocytes and SSCs, were shown to settle on the basal membrane of the recipient seminiferous tubules and form SSC colonies with expression of MAGEA, GAGE, UCHL1, SALL4, UTF1, and LIN28. The colonization efficiency was approximately 6%. No human Sertoli cells were detected in the recipient mouse testes.

**Conclusion:**

Xenotransplantation, without *in vitro* propagation, of testicular cell suspensions from infant boys with cryptorchidism resulted in colonization of mouse seminiferous tubules six to nine weeks post-transplantation. Spermatogonial stem cell-based transplantation could be a therapeutic treatment for infertility of prepubertal boys with cryptorchidism and boys diagnosed with cancer. However, more studies are required to investigate whether the low number of the transplanted SSC is sufficient to secure the presence of sperm in the ejaculate of those patients over time.

## Introduction

During the last decades, improved diagnostics and cancer treatments of children have resulted in more long-term survivors (1). However, chemotherapy and irradiation can lead to subfertility and sterility, which have serious negative effects on the life quality of survivors (2, 3). In post pubertal boys and men, semen cryopreservation prior to gonadotoxic therapy is the standard method for fertility preservation. However, this is not an option for prepubertal boys who are unable to provide a semen sample (4). Currently, cryopreservation of testicular tissue before gonadotoxic treatment, malignant and non-malignant diseases, is the only clinical method available to potentially preserve the fertility for prepubertal boys (5–7). Prepubertal testicular tissue cryopreservation (TTC) is an experimental method that preserves spermatogonial stem cells (SSCs), which are sperm progenitors that can potentially be used to restore spermatogenesis and ultimately produce spermatozoa in adulthood (8). Further, prepubertal boys treated for bilateral cryptorchidism may experience reduced sperm concentrations in adulthood, although the risks are attenuated by early orchidopexy (9). Nonetheless, around 17% to 25% of men with azoospermia have a history of cryptorchidism showing that cryptorchidism compromises fertility outcome, and in early childhood TTC performed at orchidopexy may be considered for more severe cases (10–12).

Spermatogonial stem cells appear at 3 to 12 months of age in boys usually in connection with the mini-puberty, and develop from gonocytes, which are mostly present during fetal and early neonatal life (13–15). Spermatogonial stem cells consist of A_dark_ (AdS) and A_pale_ spermatogonia. A_dark_ spermatogonia are phenotypically defined as spherical or ovoid cells with dark, dense chromatin in the nuclei frequently with a rarefaction zone, and are considered as the SSC reservoir (16). Physiologically, SSCs are thought to maintain the balance of self-renewal and differentiation to preserve the SSC pool and sustain perpetual spermatogenesis in adults. However, SSCs are a rare cell population in testis when considering “functional” capacity (17).

Transplantation of SSCs is considered a promising strategy to restore spermatogenesis and fertility in humans as proof of concept has been successfully demonstrated in rodents, domestic animals, and non-human primates (18). However, SSC transplantation in humans has not yet been successful (19). Optimal culture conditions for *in vitro* propagation of human SSCs are still lacking (20). Un-physiological *in vitro* conditions, such as enzymatic digestion, growth factors, and cytokines may result in oxidative stress and DNA damage (21, 22). In addition, zoonotic agents, including fetal bovine serum, also increase the risks for xenogeneic infections (23). Taken together, technical difficulties have hampered the translation from bench to clinic because of the lingering safety and ethical considerations. Although propagation of SSCs has been reported from infant boys under xeno-free conditions (24), the genetic and epigenetic stability of the SSCs still need to be determined before the application in clinical trials. Furthermore, the regulation for genetic and epigenetic determination also remains to be standardized.

Interestingly, in connection with transplantation of germ cells, both PGCs and gonocytes have, in addition to SSCs, shown the capability to support spermatogenesis in adult mammalian hosts post-transplantation (25, 26). Therefore, PGCs, gonocytes, and SSCs all possess the true stem cell potential and are referred to as male germline stem cells (27). Testis tissues, particularly from infant boys, contain gonocytes and SSCs, which may be used as a source of germline stem cells to restore spermatogenesis and produce sperm. Transplantation of neonatal or prepubertal germ cells from rodents has shown the capacity to colonize the host testis post-transplantation (28). However, it is not known whether human infant germ cells without *in vitro* propagation possess the capacity to colonize the testicular tubules post-transplantation.

In this study, we directly transplanted human infant testicular cells including gonocytes and SSCs into seminiferous tubules of sterilized nude mice to explore the feasibility of these stem cells to form colonies.

## Materials and Methods

### Human Testis Tissue

Testis biopsies were obtained from 11 infant boys (median age 1.3 years, range 0.5-3.5) who underwent orchidopexy at the Department of Pediatric Surgery, Copenhagen University Hospital, Rigshospitalet (**Table 1**). Ten boys were diagnosed with congenital bilateral cryptorchidism, one with congenital unilateral cryptorchidism. None of the patients received hormonal therapy or were diagnosed with other conditions. The patients have previously been included in a study evaluating parental acceptance of experimental fertility preservation in young boys (29). Prior to tissue collection, informed consent was obtained from the parents of the patients for participating in the fertility preservation program and for donating a small testicular biopsy for research purpose. Consequently, the testicular biopsy from each testis was divided into three fragments: one for clinical TTC, one for routine pathological assessment, and one for research. The testis biopsy for pathological assessment was similar to the biopsy for research. The mean weight of the 11 testicular fragments for research use was 4mg. These testicular fragments were cryopreserved according to a previous published method (30). In brief, the tissue was equilibrated in 1.5 M ethylene glycol, 0.1 M sucrose, 10 mg/ml HSA for 20 min followed by a slow freezing procedure and storage in liquid nitrogen (30).

**Table 1:** Clinical and experimental parameters of infant boys with cryptorchidism.

Patient ID	Birth weight (g)	Diagnosis	Age at orchidopexy(year)	Testis location*	Serum FSH (IU/L)	Serum LH (IU/L)	Serum inhibin B (pg/ml)
#1	3075	bilateral	0.9	abdominal	1.60	0.37	147
#2	4184	bilateral	0.7	inguinal	0.57	0.36	222
#3	4000	bilateral	1.6	supra-scrotal	0.84	0.09	72
#4	3720	bilateral	1.7	supra-scrotal	0.64	0.14	76
#5	3590	bilateral	2.5	supra-scrotal	2.5	0.38	44
#6	4272	bilateral	3.5	supra-scrotal	0.56	0.05	57
#7	3200	bilateral	1.2	supra-scrotal	0.16	0.28	143
#8	3524	bilateral	1.0	inguinal	0.89	0.05	77
#9	4110	bilateral	0.5	annulus	0.57	0.85	280
#10	4910	unilateral	1.3	annulus	1.46	0.35	41
#11	3200	bilateral	1.4	supra-scrotal	0.81	0.11	70

*For the bilateral undescended testes, testis location indicated the location of the testis biopsy used for research.

### Handling of Animals

Nude mice (Naval Medical Research Institute (NMRI)-NU, Charles River, Denmark) were housed in groups, fed pellets and water ad libitum, and kept under controlled 12-hour light/12-hour dark cycles at 20-22°C. At eight weeks of age, each testis of mice was injected with 80 μg busulfan (B2635, Sigma-Aldrich) to eliminate endogenous spermatogenesis. The busulfan was dissolved in dimethyl sulfoxide (DMSO) and delivered in a volume of 20μl through two different sites (24, 31). Xenotransplantation was performed 4-5 weeks after busulfan treatment. Both injection and xenotransplantation were performed under anesthesia using Zoletil (Virbac, France), xylazin (Scanvet, Denmark), and butorphanol (Zoetis, New Jersey). Post-operative analgesia was provided by use of buprenorphine (Reckitt Benckiser; England, UK) and carprofen (Norbrook, England, UK). Following xenotransplantation, mice were single-housed until euthanasia. Euthanasia was done by cervical dislocation.

### Histology, Cell Counting, Cryopreservation

Stieve’s fixative was used for fixation and the infant testis tissue was embedded in paraffin and cut into 2-μm sections. The sections were stained with hematoxylin and eosin (HE) and germ cell markers including podoplanin (D2-40), cluster of differentiation 99 (CD99), octamer-binding transcription factor (Oct3/4), placental alkaline phosphatase (PLAP), and KIT proto-oncogene (C-KIT) following the same protocol as previously described (32).

Spermatogonia stem cells with or without the presence of gonocytes, constituted the germ cells within the infant cryptorchid testes. The total number of germ cells that included both SSCs and gonocytes was counted. The measurements of the number of germ cells and A_dark_ spermatogonia per cross-sectional seminiferous tubules was performed as previously described (33, 34) in at least 100 and 250 cross-sectional tubules per testicular biopsy, respectively. These measurements were carried out in a blinded fashion as a prognostic effort for evaluating the fertility potential.

To estimate the number of germ cells before xenotransplantation, we analyzed the germ cells within the testicular biopsy used for pathological assessment (**Figure 2A**). All sections used for pathological assessment were visualized digitally using a NanoZoomer digital pathology scanner (Hamamatsu Photonics K.K., Hamamatsu City, Japan) and quantified (total number of germ cells/surface area) with NDP viewer software (Hamamatsu Photonics K.K.). The seminiferous tubules were examined under a magnification of 40 x, whereas the measurement of surface area (excluding tunica albuginea) was carried out using a magnification of 5 x. All digital measurements were carried out blinded by two investigators and the final estimation of the germ cell density was presented as the mean number of germ cells/mm^3^ from six non-serial sections. The germ cell density was calculated according to the following formula:


$$\text{D}=\frac{N}{A(d-t)}$$

D, density; N, number of germ cell counted; A, area of tissue on section; d, diameter of germ cell; t, thickness of the section. A prerequisite of this formula is equal distribution of cells in the section. The mean diameter of germ cells was measured from 10 germ cells per patient and only germ cells with the nucleoli visible were included for measurement. Within our samples, the diameter of the germ cell was 14 ± 1 μm (mean ± SD). The number of human germ cells prepared for each recipient was: mean weight of 11 testicular biopsies multiplied by the germ cell density (D).

The biopsies for research were placed in McCoy 5A medium (modified 22330-021, Gibco, Life Technologies, Paisley, UK) immediately after surgery for transportation to the laboratory (10 min. transport) where TTC was performed (30). The testis biopsy used for counting derived from the same biopsy which was used for xenotransplantation.

### Cell Isolation and Xenotransplantation

**Figure 1:**
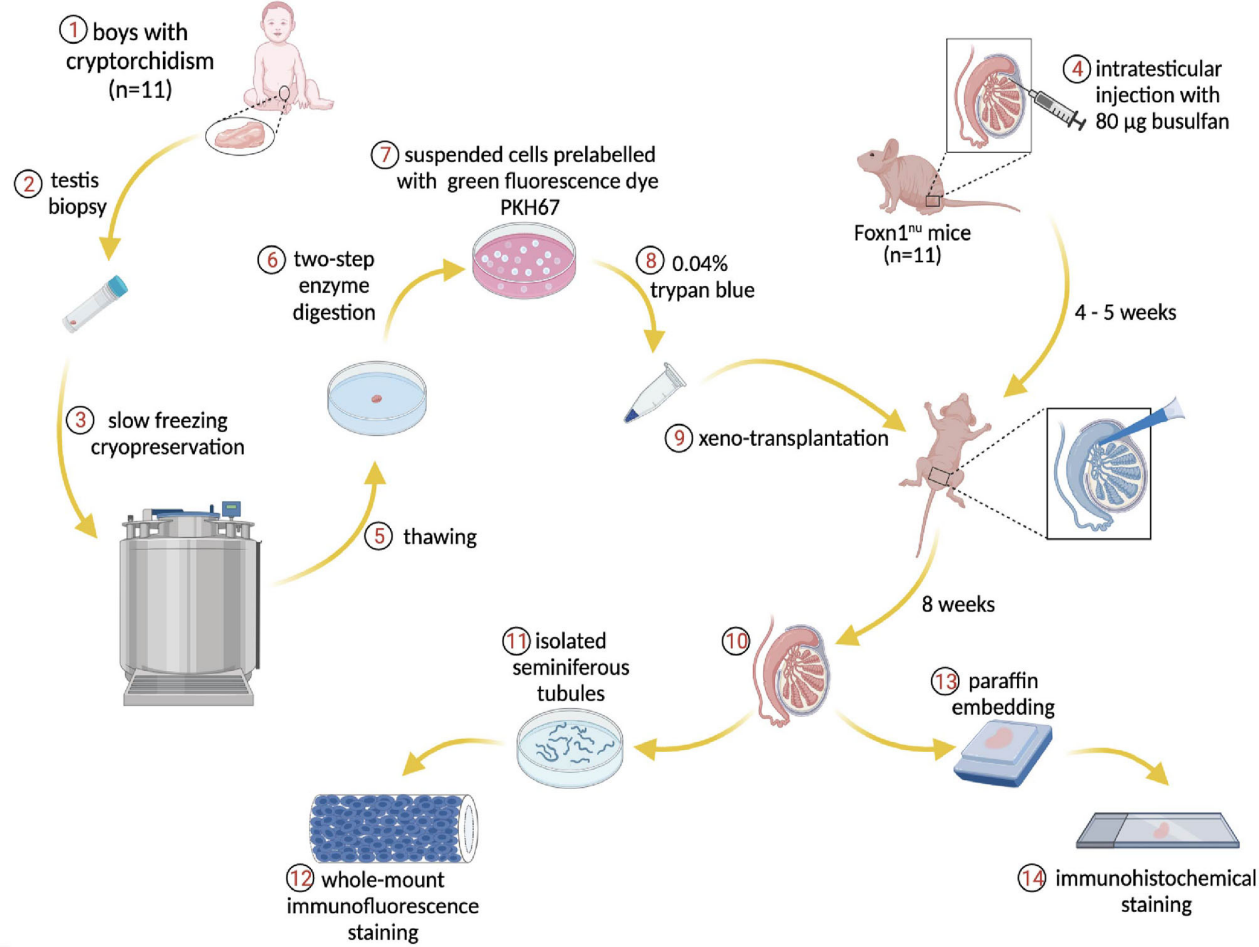
Schematic overview of the experiment. Donor testis biopsies from infant boys with cryptorchidism underwent a frozen-thawed procedure and were dissociated through two-step enzymatic digestion. The suspended cells were prelabeled with PKH67 for tracing human cells within the recipient mouse seminiferous tubules post-transplantation. The endogenous spermatogenesis of recipient mice was eliminated by busulfan treatment. The human testicular cells were injected into the mouse tubules and the transplantation process was visible *via* trypan blue. Eight weeks later, the testes were harvested for immunostaining and further analysis.

Frozen testicular biopsies were thawed by three steps for 10 min in each medium: 1) 0.75 M ethylene glycol, 0.25 M sucrose in PBS, and 10 mg/ml Human serum albumin (HSA) (CSL Behring, Germany); 2) 0.25 M sucrose in PBS and 10 mg/ml HSA; 3) PBS and 10 mg/ml HSA according to a previously published method (30). Immediately after thawing, testicular biopsies were digested in α-MEM media supplemented with 2 mg/ml Collagenase type I (Worthington), 2 mg/ml Hyaluronidase type II (Sigma), 2 mg/ml Trypsin TRL3 (Worthington), and 16 μg/ml DNase I (Sigma) for 15 min at 37°C. After centrifugation, digested tissues were resuspended in Collagenase type I, Hyaluronidase type II, and DNase I at conditions similar to the first digestion and incubated for 30 min at 37°C. Human serum albumin 10 mg/ml was used to quench the enzyme activity. The cell suspensions were filtered through a 70 μm and subsequently a 40 μm strainer. Before transplantation, suspended cells were prelabeled with a green fluorescent dye PKH-67 (Sigma) according to manufacturer instructions (35). At transplantation, 15 μl containing 10^5^ of testicular cells and 0.04% trypan blue (Sigma) were injected into the seminiferous tubules of recipient testis through the efferent duct (**Figure 1**).

### Immunostaining

At 6 to 9 weeks post-transplantation, recipient testes were harvested and cut into a large (4/5 of the intact testis) and a small piece (1/5 of the intact testis). The large piece was used for analyzing human testicular cell colonization by whole-mount immunofluorescence in a three-dimensional arrangement. The small piece was fixed in formalin, embedded in paraffin, and cut into 5-μm sections for immunohistochemical staining. Moreover, immunohistochemistry was used to identify human cells using anti-human nuclear antigen antibody (anti-H). For the whole-mount immunofluorescence staining, tunica albuginea of the recipient testis was removed, and the seminiferous tubules were gently separated and digested with DNase I (1 mg/ml) and Collagenase IV (1 mg/ml) in Hanks’ balanced salt solution (HBSS). Dispersed tubules were fixed in 4% formaldehyde and washed in PBS. Tubules were incubated in 10% methanol and blocked with 2% bovine serum albumin and 0.5% Triton X-100 in PBS.

Histological sections were incubated overnight at 4°C with the following primary antibodies (**Supplementary Table 1**), anti-melanoma antigen genes-A mouse monoclonal antibody (MAGEA), anti-G antigen (GAGE) mouse monoclonal antibody. MAGEA and GAGE are cancer/testis-associated proteins encoded by gene clusters located on the X chromosome and expressed in spermatogonia and primary spermatocytes in adult testis and also in migrating primordial germ cells in human embryo (36–38). We also used anti-ubiquitin carboxyl-terminal hydrolase L1 mouse monoclonal antibody (UCHL1) which is a spermatogonial marker (20, 39); anti-Sal-like protein 4 mouse monoclonal antibody (SALL4) is a member of *sal*-gene family of transcription factors and a conserved marker of spermatogonia (40, 41); anti-undifferentiated embryonic cell transcriptional factor 1 mouse monoclonal antibody (UTF1) is a spermatogonial marker (42); and anti-LIN28 mouse monoclonal antibody (LIN28) is a RNA-binding protein expressed in gonocyte of fetal testis and spermatogonia of postnatal testis (43). Moreover, anti-SOX9 rabbit polyclonal antibody (SOX9) was used as a Sertoli cell marker, anti-cytochrome P450 17A1 goat polyclonal antibody (CYP17A1) was used as a Leydig cell marker. After wash in Tris-buffered saline with Tween20^®^ (Sigma)(TBST), the slides were incubated with secondary antibodies anti-mouse Alexa 594/anti-rabbit Alexa 594/anti-goat Alexa 568 (1:500, Jackson ImmunoResearch) for 1 hour at room temperature (RT). After nuclear staining with 4’,6 - diamidino-2-phenylindole (DAPI), the seminiferous tubules were mounted on slides with antifade reagent (Invitrogen). The green cells (human cells prelabeled with PKH67) were counted and cells at least 150μm apart were considered as different colonies. The colonization efficiency was calculated as no. human germ cell colonies identified/no. human germ cells injected. The testes from recipients no.1 to no.8 were all harvested 8 weeks post-transplantation, whereas no.9, no.10, no.11 were collected at 7 weeks, 6 weeks, 9 weeks post-transplantation, respectively (**Table 2**).

**Table 2:** Colonization efficiency of germ cells from infant boys with cryptorchidism.

Recipient	Testis	Patient ID	No. human germ cells prepared^a^	% human cells injected to recipient^a^	No. human germ cells actually injected^c^	No. human germ cell colonies found after transplantation^b^	No. human germ cells found after transplantation^b^	% mean colonization efficiency^c^
1	left	#1	56798	5 - 15	2840 - 8520	43	256	1.1
	right			5 - 15	2840 - 8520	51	303	
2	left	#2	43097	5 - 15	2155 - 6465	21	128	0.6
	right			5 - 15	2155 - 6465	16	96	
3	left	#3	10703	10 - 20	1070 - 2141	19	112	6.3
	right			5 - 15	535 - 1605	90	542	
4	left	#4	3681	15 - 25	552 - 920	19	112	3.8
	right			25 - 35	920 - 1288	51	303	
5	left	#5	6128	20 -30	1226 - 1838	85	511	9.0
	right			5 - 15	306 - 919	56	335	
6	left	#6	19841	5 - 15	992 - 2976	77	463	3.0
	right		19841	100	19841	149	894	
7	left	#7	11303	10 - 20	1130 - 2261	85	693	3.1
	right			60 - 70	6782 - 7912	37	187	
8	left	#8	3025	5 - 15	151 - 454	75	702	18.6
	right			30 - 40	908 - 1210	43	427	
9	left (7 weeks)	#9	14696	5 - 15	735 - 2204	10	96	0.5
	right (7weeks)			5 - 15	735 - 2204	0	0	
10	left (6 weeks)	#10	7959	30 - 40	2388 - 3184	98	1127	1.8
	right (6 weeks)			100	7959	1	10	
11	left (9 weeks)	#11	5361	60 - 70	3217 - 3753	86	942	6.4
	right (9 weeks)			10 - 20	536 - 1072	74	817	

Recipient testis no. 1 to no. 8 were harvested 8 weeks after xenotransplantation; no.9 to no.11 were harvested 7, 6, 9 weeks after xenotransplantation.

^a^estimated number; ^b^counted number; ^c^calculated number.

No. human germ cells actually injected = No. human germ cells prepared * % human cells actually injected to recipient; Mean colonization efficiency % = (No. human germ cell colonies obtained after transplantation/No. human germ cells actually injected) * 100.

For the immunohistochemical analysis, slides were deparaffinized and rehydrated with series of graded ethanol, followed by antigen retrieval in TEG buffer (10 mM Tris, 0.5 mM ethylene glycol-bis (2-aminoethylehter)-N, N, N’, N’-tetraacetic acid (EGTA), pH 9.0) in boiling water for 30 min. Endogenous peroxidases were blocked by 0.5% H_2_O_2_ for 15 min, and non-specific binding was blocked with 4% bovine serum albumin (BSA) and 5% donkey serum (DS) for 30 min at RT. The slides were incubated at +4°C overnight with primary antibodies, MAGEA/GAGE/UCHL1/SALL4/UTF1/LIN28/Sox9/CYP17A1 and anti-H (**Supplementary Table 1**). After wash in TBST, the secondary antibody was added, donkey anti-mouse/rabbit/goat antibody (Dako) horseradish peroxidase for 30 min at RT, visualized with 3,3’-diaminobenzidine tetrahydrochloride (Dako) for 1-2 min, counterstained with Mayer’s hematoxylin and mounted with Pertex (Histolab). All the slides were analyzed, and images were taken under a microscope with a digital camera (Leica).

### Statistical Analysis

Individual values were shown as mean ± standard deviation (SD). GraphPad Prism version 8.0 was used for statistical analyses. The data of colonization efficiency, hormone levels, and age followed a normal distribution. Correlations between colonization efficiency and hormone levels/age were tested by Pearson correlation coefficient. The multiple comparison was performed with Kruskal – Wallis test among colonization efficiency at different weeks. *P* values less than 0.05 were considered statistically significant.

## Results

### Characterization of Infant Testes

**Figure 2:**
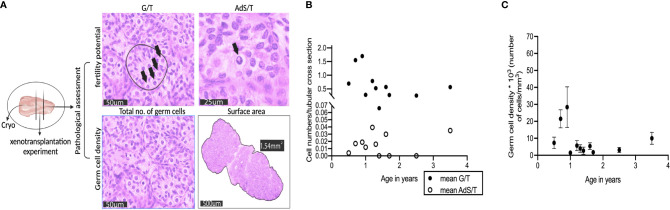
Characterization of donor testis biopsies from infant boys with cryptorchidism. **(A)** The testis biopsies were cut into three parts. The bigger biopsy was cryopreserved in a biobank for fertility preservation, and the size of the other two pieces was similar, one for pathological assessment of the fertility potential and germ cell density, the other for experimental use. All four figures were from a testis biopsy from a 0.9-year-old infant boy with bilateral cryptorchidism (Patient ID #1). Germ cells per tubular cross section (G/T), A_dark_ spermatogonia per tubular cross section (AdS/T). G/T figure: four germ cells (black arrow) within one seminiferous tubule (black circle), scale bar, 50μm; AdS/T figure: an A_dark_ spermatogonium (black arrow), scale bar, 25 μm; total number of germ cells figure: scale bar, 50 μm; surface area figure: 1.54 mm^2^, scale bar, 500 μm. **(B)** The number of germ cells (G/T) and A_dark_ spermatogonia (AdS/T) per tubular-cross section from 11 testis biopsies, x-axis represents age in years. **(C)** Germ cell density was the total number of germ cells per mm^3^ on tubular sections from 11 testis biopsies, x-axis represents age in years. Dots represent the mean value; error bars represent standard deviation (SD).

Spermatogonia were located on the basal membrane, while the gonocytes were present in the center of the tubules. The number of germ cells per tubular cross-section (G/T) ranged from 0.07 to 1.70 among patients while some testis samples were lacking A_dark_ spermatogonia (**Figure 2B**). According to the formula of germ cell density, we found that the initial mean germ cell density (before xenotransplantation) was from 1513 to 28399 cells per mm^3^ (**Figure 2C**). The weight of the testicular tissues before cryopreservation was 4 ± 3 mg. Combining the germ cell density and the weight of the testicular biopsies, we estimated the number of human germ cells prepared for each recipient (**Table 2**).

**Figure 3:**
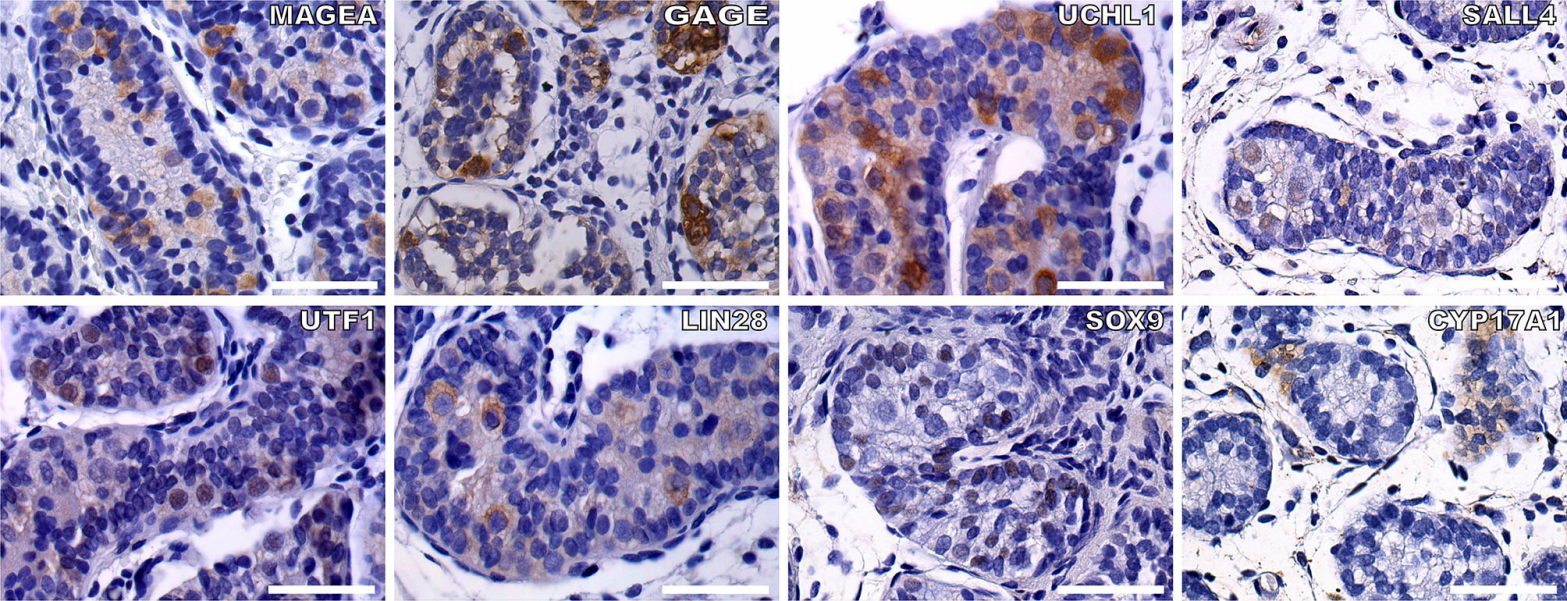
To check the feasibility of the germ cell and somatic cell markers, we did IHC staining of germ cells and Sertoli cells within the seminiferous tubules from two infant boys with cryptorchidism. Germ cell markers included: MAGEA, GAGE, UCHL1, SALL4, UTF1, and LIN28, the positive staining of germ cells within the seminiferous tubules (brown). Sertoli cells were indicated by SOX9 (brown). CYP17A1 stained the Leydig cells (brown). The nuclei were stained by hematoxylin (blue). Scale bar, 50 μm.

To evaluate the specificity of the immunohistochemical markers, reference testis tissues from two infant boys with cryptorchidism were used. MAGEA, GAGE, UCHL1, SALL4, UTF1, and LIN28 were all expressed in germ cells located in the lumen and/or on the basal membrane of the seminiferous tubules. SOX9 was expressed in the nuclei of the Sertoli cells (**Figure 3**).

### Colonization of Recipient Testes by Human Germ Cells

We found that human infant germ cells could form colonies within the recipient seminiferous tubules 8 weeks post-transplantation. Based on the appearance of the seminiferous tubules after histological staining, we were unable to identify human germ cells in meiosis (i.e., from preleptotene onwards). The actual location of human germ cells in the seminiferous tubules were on the basal membrane.

### Whole-Mount Analysis of Germ Cell Colonization Efficiency

**Figure 4:**
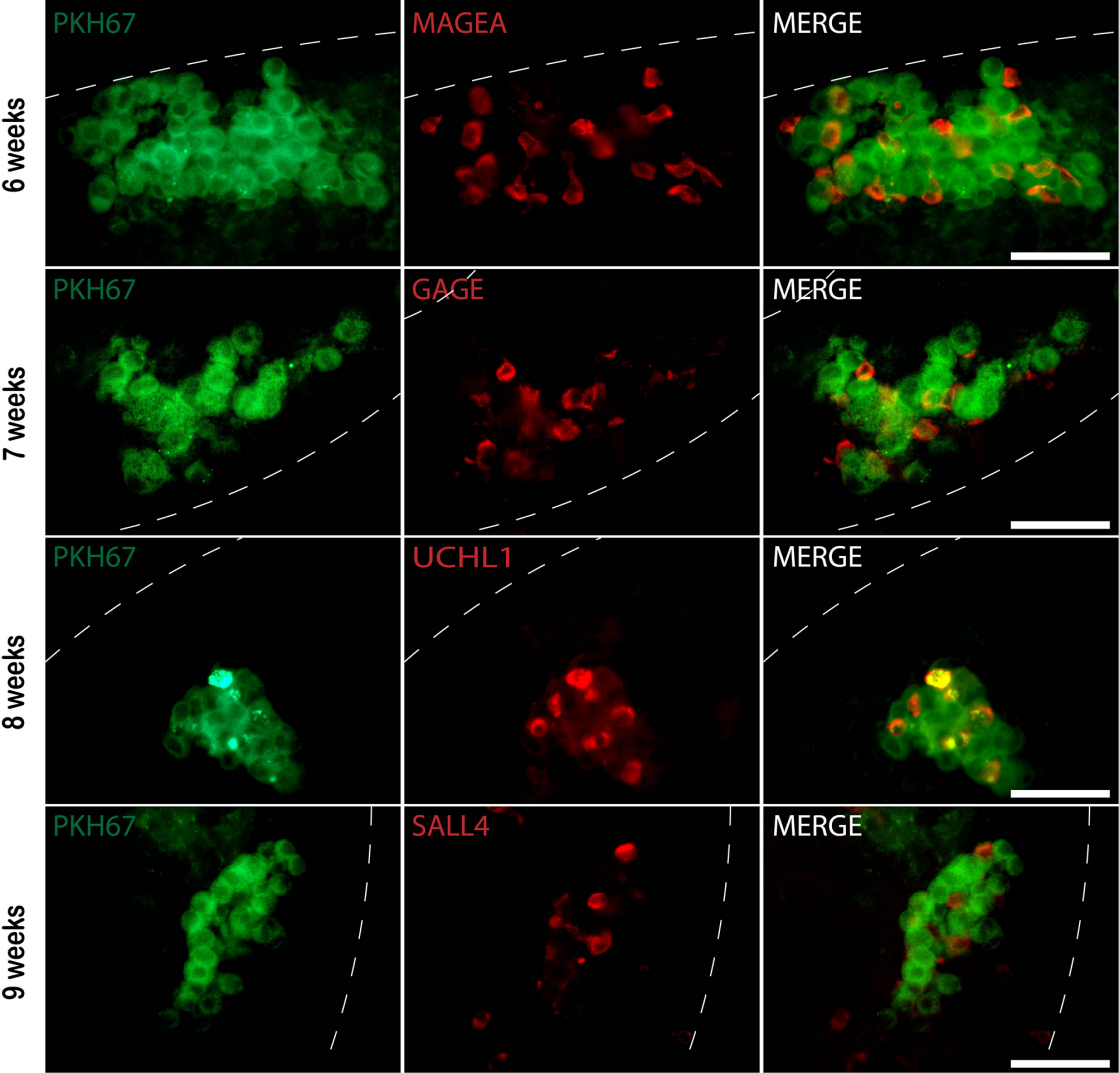
Whole-mount immunofluorescence (IF) staining of recipient mouse seminiferous tubules xenotransplanted with donor human testicular cells prelabeled with PKH67. Six to nine weeks post-transplantation, PKH67 cells (green) were near the basement membrane of the mouse seminiferous tubules (white dotted lines) and co-stained with different germ cell markers (red). 6 weeks: Donor testis biopsy was from a 1.3-year-old boy (Patient ID #10). PKH67-positive cells (green) co-expressing germ cell marker MAGEA (red) within the recipient mouse tubule. 7, 8, and 9 weeks: Donor testis biopsies were from boys at 0.5, 2.5, 1.4 years old, respectively (Patient ID #9, #5, #11). PKH67-positive cells (green) were found inside the recipient tubules and expressed GAGE (red), UCHL1 (red), and SALL4 (red). The nuclei were stained by DAPI (blue). Scale bar, 50 μm.

**Figure 5:**
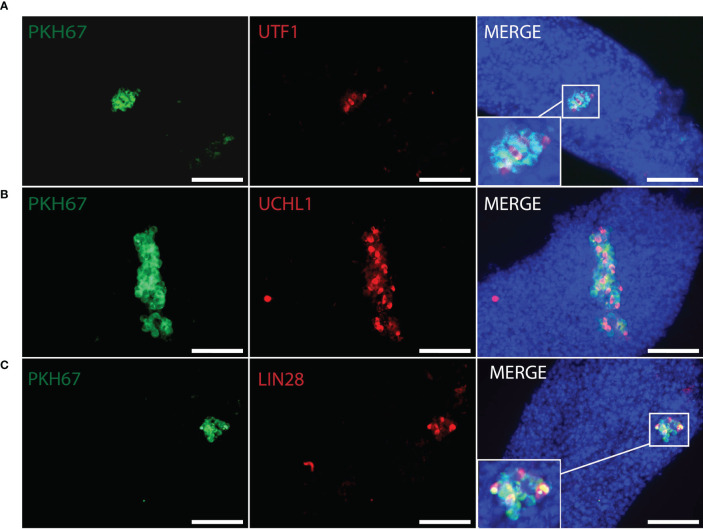
Whole-mount detection of human germ cells xenotransplanted into recipient mouse seminiferous tubules by different germ cell markers. PKH67-positive cells (green) indicated human cells co-stained with **(A)** UTF1 (red), **(B)** UCHL1 (red), **(C)** LIN28 (red) within the recipient mouse tubules. Corresponding to figures **(A–C)**, donor testis biopsies were from Patient ID #11, #5, #5. DAPI (blue) for nuclear staining. Scale bar, 100 μm.

**Figure 6:**
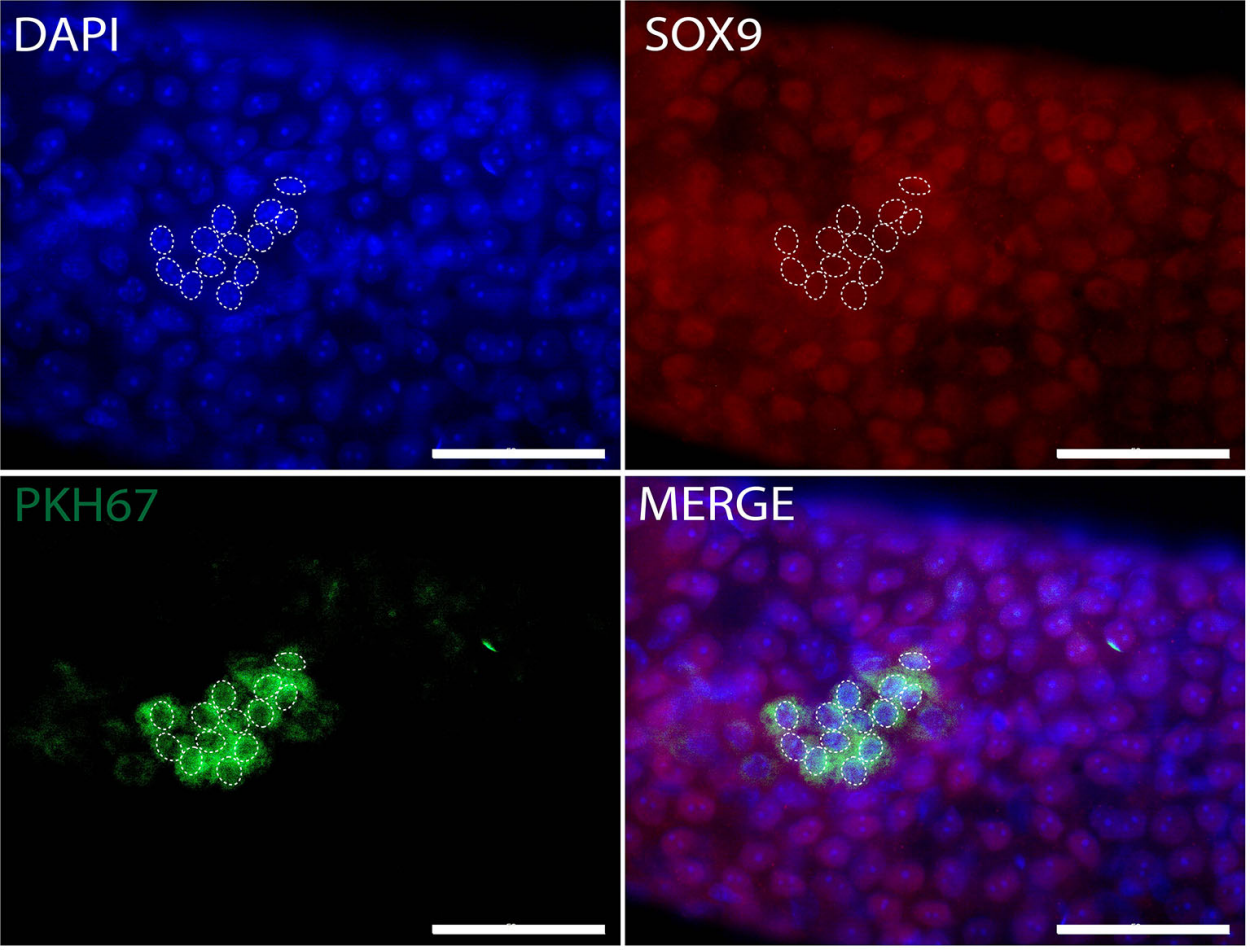
Whole-mount detection of human Sertoli cells xenotransplanted into recipient mouse seminiferous tubules. PKH67-positive cells (green) indicating human cells without expression of Sertoli cell marker SOX9 (red). White dotted circles: PKH67-positive cells. The nuclei were stained by DAPI (blue). Scale bar, 50 μm.

Whole-mount immunofluorescence staining identified human germ cells positive for both PKH67 and the germ cell markers, MAGEA, GAGE, UCHL1, SALL4, UTF1, and LIN28 (**Figures 4**, **5**). By changing the focal plane of the microscope, we could observe these human germ cells on the outer layer of the tubules indicating that they were located on the basement membrane of the tubules. The Sertoli cell marker SOX9 was not expressed in the nuclei of the green human cells, indicating that the colonies did not contain any Sertoli cells (**Figure 6**). We found one single human Leydig cell stained by CYP17A1 that survived in the recipient testis (**Supplementary Figure 1**). Thus, we considered the cells within the colonies were all germ cells. Human PKH67-positive cells stained green but not all germ cells were detected using each of the applied markers and showed that different phenotypes of human germ cells survived transplantation (**Figures 4**, **5**).

The mean colonization efficiency was 6.4% at nine weeks post-transplantation, and 1.8% and 5.7% at six weeks and eight weeks, respectively (**Table 2**), which was not significantly different. We injected the full volume of 15μl cell suspensions into the right side of recipient testis no.6 and no.10. However, the colonization efficiency (right side of recipient testis no.6) was only 1%. Almost all seminiferous tubules within the right-sided recipient testis of no. 10 became atrophic and solidified at six weeks post-transplantation and only 1 colony was found from the viable tubules. There was a positive correlation between the number of human germ cells actually injected and human germ cell colonies obtained eight weeks post-transplantation (r = 0.50, *P =* 0.048). (**Table 2**). No correlation was found between colonization efficiency and clinical parameters (serum hormones, age) (**Tables 1**, **2**).

### Immunohistochemical Analysis

**Figure 7:**
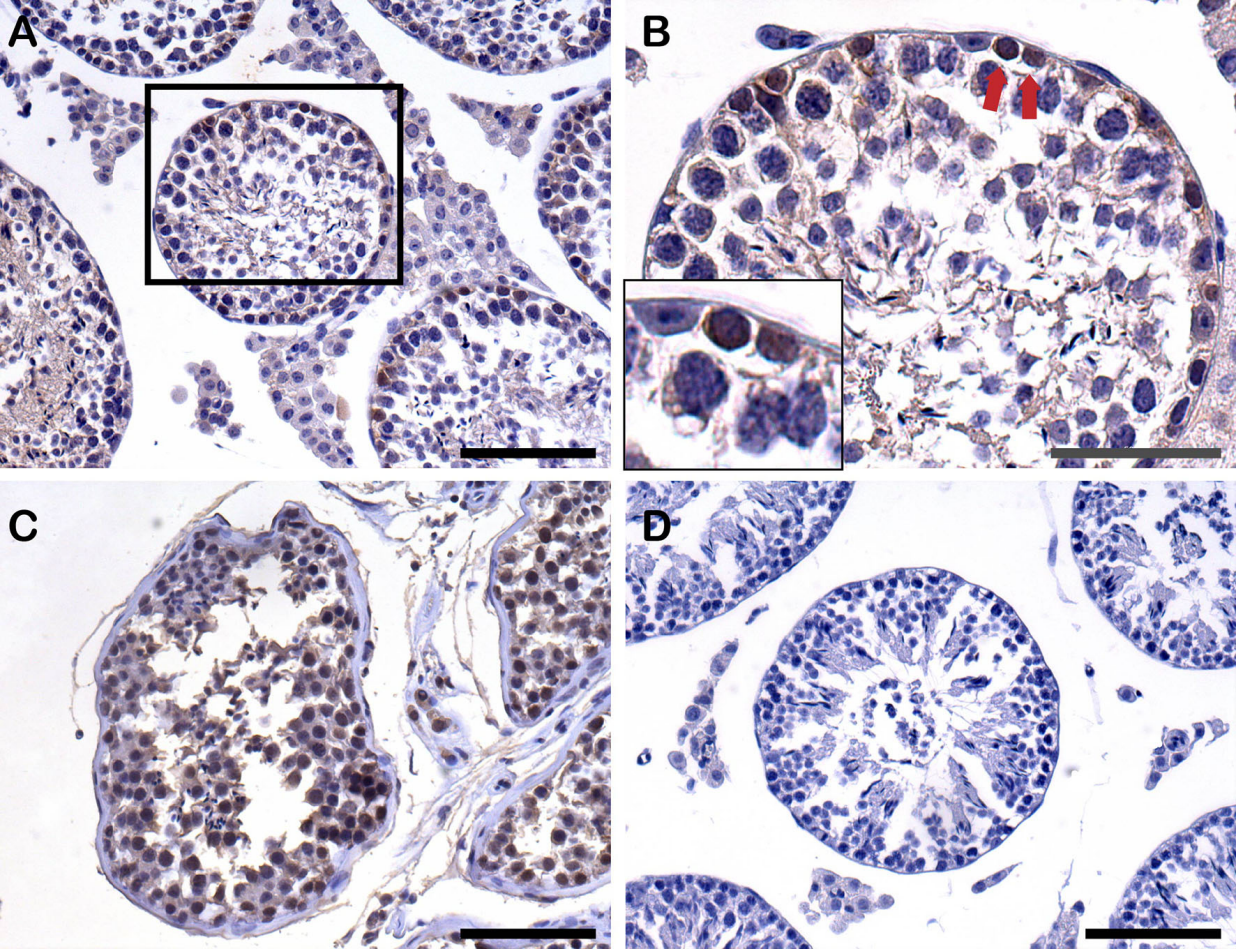
Detection of human cells by anti-human nuclear antigen antibody (anti-H) by immunohistochemistry (IHC) staining on paraffin-embedded testis sections from recipients after xenotransplantation. **(A)** Human cells (anti-H positive cells) settled down on the basement membrane of recipient seminiferous tubules 8 weeks post-transplantation. Scale bar, 100 μm. **(B)** Higher magnification of recipient seminiferous tubule with red arrows indicating the anti-H positive human cells on the basement membrane. Scale bar, 50 μm. **(C)** Positive control of anti-H from normal human adult testis tissue. Scale bar, 100 μm. **(D)** Negative control of anti-H from mouse testis tissue. Scale bar, 100 μm.

The anti-human nuclear antigen antibody expressed on the recipient testis sections also identified human cells and proved to survive human cells 8-weeks post-transplantation. The human cells were positioned on the basal membrane of the recipient seminiferous tubules (**Figure 7**).

## Discussion

This study demonstrated that germ cells from infant boys with cryptorchidism can colonize the recipient mouse testes and survive six to nine weeks post-transplantation without purification or propagation before transplantation. Our study showed that human germ cells were located in the niche on the basal membrane of the recipient seminiferous tubules and able to proliferate but failed to progress in sperm development. These results are in line with a previous report in which no spermatogenesis was detected after xenotransplantation human testicular cells for up to six months post-transplantation (19).

We showed that the survived human cells prelabeled with the dye PKH67 were SSCs with different phenotypes expressing MAGEA, GAGE, UCHL1, SALL4, UTF1, and LIN28. This confirms and extends a previous finding, that after transplantation of flow cytometry sorted human spermatogonia, different phenotypes of spermatogonia can result (41, 44). Thus, we confirmed that not only one human phenotype of infant SSCs has the capacity to colonize the recipient seminiferous tubules and settle in the niche. Therefore, it is likely that a variety of different phenotypes of SSCs may be used for transplantation in future clinical studies for fertility restoration in adult men.

Direct transplantation of infant germ cells without *in vitro* propagation has the advantages of reducing possible genetic and epigenetic changes due to culture conditions improving its safety and also, it is easier to be applied in a clinic in the future. A prerequisite for performing transplantation of only enzyme digested testis tissue is a sufficient number of transplanted germ cells settle in the proper niche to sustain renewed spermatogenesis. Since data suggest that spontaneous spermatogenesis may occur after chemotherapy causing a severe depletion of SSCs (45), it may be hypothesized that only a few human SSCs, once in the proper microenvironment, may be sufficient for spermatogenesis to recover over time. Thus, after some chemotherapy regimen conditions, direct transplantation of even a small number of SSCs could result in sperm production during a long-term *in vivo* propagation of SSCs. Currently, it is unknown how fast spermatogenesis could potentially be re-established, but a considerable time may be required. However, even if some years were required for spermatogenesis to take place, this could still be achieved by transplanting applied to these boys as adolescents allowing them to be fertile as the normal age of fatherhood.

We were unable to detect human Sertoli cells at eight weeks following transplantation in the recipient testis. The absence of human Sertoli cells could be due to the fact that the recipient mouse Sertoli cells were not eliminated by the busulfan treatment, that was used to deprive endogenous germ cells to provide space for donor germ cells. Therefore, it is hypothesized that there was no extra space for the human Sertoli cells to settle down in the mouse tubules. Previously, studies have reported that after removing recipient endogenous Sertoli cells, donor Sertoli cells may colonize the recipient’s seminiferous tubules (46, 47). Another explanation could be that the human Sertoli cells were phagocytized by the recipient Sertoli cells or macrophages. This is substantiated by results showing that donor germ cells which failed to attach to the basal membrane were phagocytized by the recipient Sertoli cells and that released sperm could be engulfed by macrophages (48). However, one study on transplantation of bovine testicular cells into mouse testes showed that some bovine Sertoli cells survived in mouse tubules two months post-transplantation (49). In our study, we did not stain all the tubules for somatic cell markers, and we cannot exclude that there could be a few donor somatic cells surviving in other parts of the testis. As we identified one single human Leydig cell that survived within mouse testis post-transplantation, further studies are needed to investigate whether human Sertoli cells and Leydig cells may survive in larger numbers in the recipient testis post-transplantation.

Human germ cells migrated to the basement membrane of the mouse seminiferous tubules, settled, and formed colonies. According to previous studies, one colony is often formed from just one SSC (50, 51). In our study, the number of human germ cell colonies formed after xenotransplantation was presumed to be equal to the number of survived human SSCs with proliferative activity. We found approximately 55 colonies per 10^5^ testicular cells at eight weeks post-transplantation. Valli and colleagues reported four to nine colonies per 10^5^ cells eight weeks post-transplantation. To enrich the colonization efficacy, they used fluorescence-activated cell sorting to sort out different phenotypes of SSCs followed by xenotransplantation and reported around 50 colonies per 10^5^ cells within the recipient tubules two months post-transplantation (44). Thus, they achieved a 12-fold enrichment of colonies compared to the unsorted population (44). We reached colonization efficacy similar to the sorted fraction in their study. However, the colonization efficacy may be related to the age of the boys, which in our study was 0.5 – 3.5 years, while postpubertal donors (age 14 – 50 years) were used in Valli’s study.

If the direct transplantation of a few human SSCs is not sufficient, then propagation of SSCs prior to transplantation is necessary. In order to evaluate the magnitude of SSC propagation required, Sadri et al. calculated that SSCs within a volume of 200ul of a prepubertal testis biopsy were required to be increased by 65-fold in order to colonize an adult normal-sized 13ml testis after SSCs transplantation (52, 53). According to the colonization efficiency of SSCs from animal auto-transplantation models, they assumed that the efficiency for human SSCs auto-transplantation is at least 5% and therefore a 1300-fold increase of human SSCs is required for a sufficient number of cells to justify transplantation. In our study, the volume of infant testis biopsy was approximately 4ul and the mean colonization efficiency was around 6% eight weeks post-transplantation. According to the calculation method from Sadri et al. (52), the number of SSCs within a 4ul biopsy from an infant testis is required to increase 3250-fold to contain sufficient cells to colonize an adult testis after transplantation. The concentration of SSCs differs between an infant and an adult testis and our results showed that infant testis biopsies had about 10-fold enrichment of SSC colonies compared to the biopsies from adult donors. Therefore, an approximate 5400-fold (3250-fold/10/6%) enrichment of SSCs would be necessary for sufficient repopulation of an adult testis. Thus, although these calculations are subject to several uncertainties, these results suggest that propagating SSCs *in vitro* is a necessary step.

In addition, colonization efficiency was crucial for obtaining sufficient SSC to repopulate the recipient testis. We found that patient no. 8 (age 1.0 year) where the least number of cells have been injected, the colonization efficiency was highest. In contrast, the lowest colonization efficiency was observed for patient no.2 and no.9 (age 0.7 and 0.5 years, respectively). These three patients were among the four youngest patients in our study. Therefore, our data do not support the age of the patient as a determining factor for colonization efficiency.

Successful spermatogenesis following transplantation of early-stage donor germ cells may also be related to the age of recipients. Mouse PGCs could produce spermatozoa in neonatal recipient mice (54), while macaque PGCs failed to achieve spermatogenesis in adult macaque recipients (25). For the future auto-transplantation of human SSCs from the infant or early prepubertal testis, the age of male recipients undergoing SSCs auto-transplantation could also be important.

It is a limitation of current study that the viability of different types of testicular cells immediately before xenotransplantation is not measured, including viability of germ cells (i.e., after cryopreservation, thawing, and enzymatic digestion), and this may affect the results obtained after xenotransplantation to the recipient mice. However, we do not consider this to be a major factor influencing the results (partly based on own results).

In theory, the use of mouse monoclonal antibodies in mouse tissue is usually followed by difficulties in getting robust results. However, in this study the antibodies were used to detect human cells in IF, which in most instances were PKH67 positive cells. In addition, we checked that the anti-human nuclear antigen antibody (anti-H) showed no cross-reaction with mouse testicular cells (**Figure 7D**). Other studies have used a similar approach to ours (19, 55).

Collectively, our data suggest that human germ cells consisting of gonocytes and SSCs from infant boys with cryptorchidism are capable of colonizing mouse seminiferous tubules eight weeks post-transplantation. With the successful re-establishment of spermatogenesis following SSC auto-transplantation in rodents and non-human primates, it is expected that auto-transplantation of human germ cells will also result in sperm generation. Further studies to determine the minimum number of SSCs and the timeframe required for the initiation and establishment of spermatogenesis following SSC auto-transplantation are needed in order to evaluate the potential clinical application of direct SSC transplantation. However, it is possible that the number of donor SSCs needs to be enriched to improve chances for successful re-initiation of spermatogenesis.

## Data Availability Statement

The original contributions presented in the study are included in the article/**Supplementary Material**. Further inquiries can be directed to the corresponding author.

## Ethics Statement 

The studies involving human participants were reviewed and approved by Regional Ethics Committee of Copenhagen (No. H-2 2012-060.anm.37655). Written informed consent to participate in this study was provided by the participants’ legal guardian/next of kin. The animal study was reviewed and approved by Animal Experiments Inspectorate (approval number 2020-15-0201-00549).

## Author Contributions

DW, LD, LM, JF, EH, EC-L, DC, JT, and CA designed the experiments. DW, SH, LD, and SP performed the experiments. DW, SH, EN, LD, SP, LM, DC, JT, and CA performed data analysis and interpretation. DW wrote the manuscript. All authors contributed to the article and approved the submitted version.

## Funding

This research was supported by the University Hospital of Copenhagen, Rigshospitalet, and the ReproUnion network (ReproUnion 2.0) financed by the European Union, Interreg V KS, and supported by Vissing Fonden (519140 AHO/PPT) and the Danish Child Cancer Foundation (2021-7395).

## Conflict of Interest

The authors declare that the research was conducted in the absence of any commercial or financial relationships that could be construed as a potential conflict of interest.

## Publisher’s Note

All claims expressed in this article are solely those of the authors and do not necessarily represent those of their affiliated organizations, or those of the publisher, the editors and the reviewers. Any product that may be evaluated in this article, or claim that may be made by its manufacturer, is not guaranteed or endorsed by the publisher.

## Supplementary Material

Supplementary Figure 1 Whole-mount detection of human Leydig cells into recipient mouse seminiferous tubules following xenotransplantation. One PKH67-positive cell (green) indicating one human cell co-stained with Leydig cell marker CYP17A1 (red) was found within the recipient seminiferous tubule. The nuclei were stained by DAPI (blue). Scale bar, 50 μm.
