# Supplementary figures and images for: Characterization and Survival of Human Infant Testicular Cells After Direct Xenotransplantation
Source: Front Endocrinol (Lausanne). 2022 Mar 10;13:853482. doi: 10.3389/fendo.2022.853482 (PMC8960121; doi:10.3389/fendo.2022.853482)

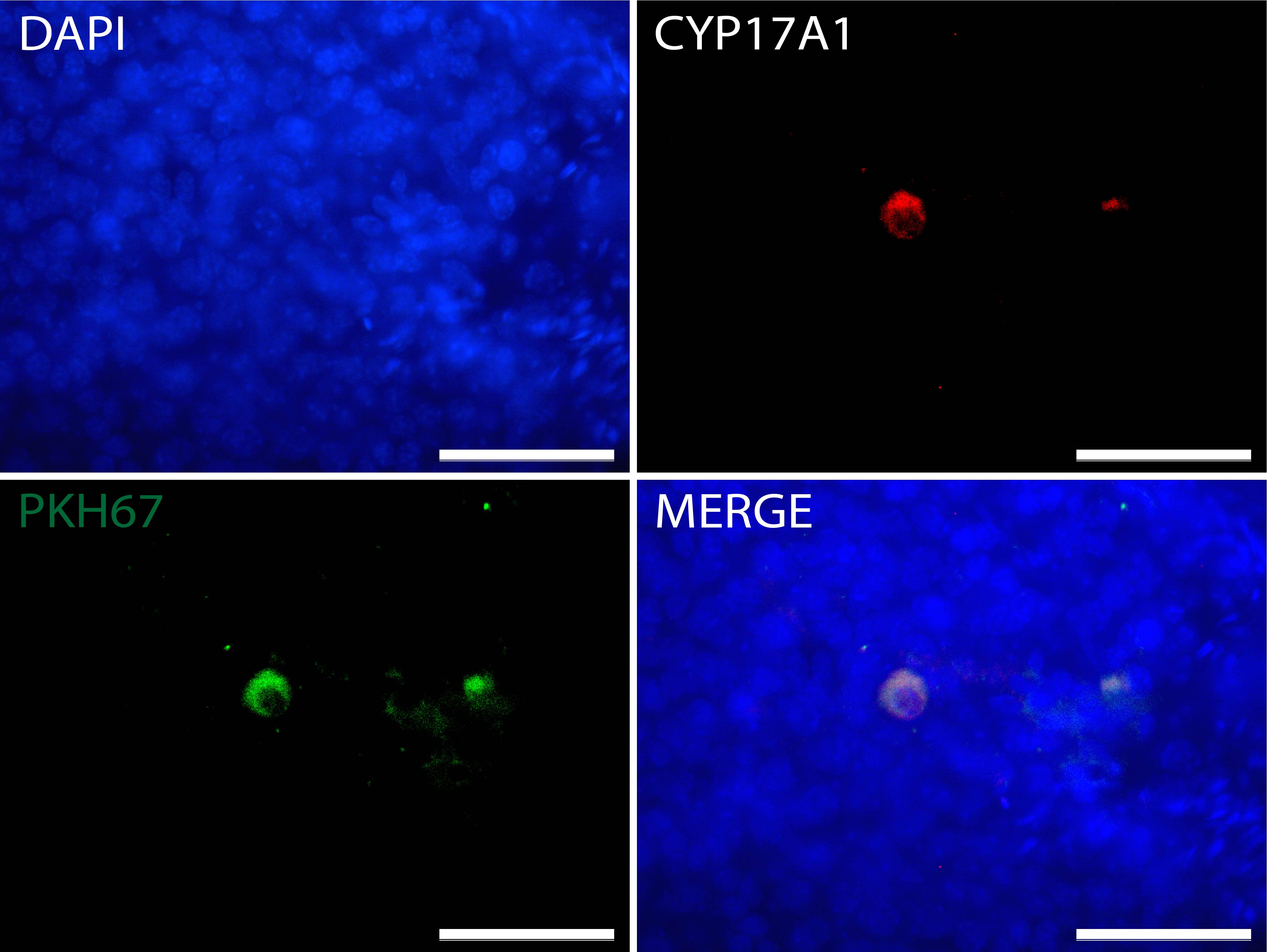

Supplement: Supplementary Figure 1 — Whole-mount detection of human Leydig cells into recipient mouse seminiferous tubules following xenotransplantation. One PKH67-positive cell (green) indicating one human cell co-stained with Leydig cell marker CYP17A1 (red) was found within the recipient seminiferous tubule. The nuclei were stained by DAPI (blue). Scale bar, 50 μm. [file Image_1.jpeg]
