# Supplementary material for: Characterization and Survival of Human Infant Testicular Cells After Direct Xenotransplantation
Source: Front Endocrinol (Lausanne). 2022 Mar 10;13:853482. doi: 10.3389/fendo.2022.853482 (PMC8960121; doi:10.3389/fendo.2022.853482)
Supplement: Supplementary file 2 [file Table_1.docx]

**Supplementary Table 1** Primary antibodies and target cells

| Antibody | Target cells | Concentration | Catalog number | Manufacturer |
| --- | --- | --- | --- | --- |
| MAGEA | spermatogonia and spermatocytes | 1:100 | sc-20034 | Santa Cruz |
| GAGE | spermatogonia | 1:100 | G13520 | Transduction |
| UCHL1 | spermatogonia | 1:100 | sc-271639 | Santa Cruz |
| SALL4 | spermatogonia | 1:100 | sc-101147 | Santa Cruz |
| UTF1 | spermatogonia | 1:100 | MAB4337 | Merck Millipore |
| LIN28 | gonocytes and spermatogonia | 1:100 | sc-374460 | Santa Cruz |
| SOX9 | Sertoli cells | 1:100 | AB5535 | Merck Millipore |
| CYP17A1 | Leydig cells | 1:200 | sc-46084 | Santa Cruz |
| anti-human nuclear antigen antibody (anti-H) | human cells | 1:50 | ab191181 | abcam |
